# Effects of Overproduction of Rubisco Activase on Rubisco Content in Transgenic Rice Grown at Different N Levels

**DOI:** 10.3390/ijms21051626

**Published:** 2020-02-27

**Authors:** Mao Suganami, Yuji Suzuki, Eri Kondo, Shinji Nishida, So Konno, Amane Makino

**Affiliations:** 1 Graduate School of Agricultural Science, Tohoku University, 468-1 Aramaki-Aoba, Aoba-ku, Sendai 980-8572, Japan; mao.suganami.p7@dc.tohoku.ac.jp (M.S.); eri.kondo.c2@tohoku.ac.jp (E.K.); shinjinishida1@gmail.com (S.N.); sou.konno.t4@dc.tohoku.ac.jp (S.K.); 2 Faculty of Agriculture, Iwate University, 3-18-8 Ueda, Morioka, Iwate 020-8550, Japan; ysuzuki@iwate-u.ac.jp

**Keywords:** Rubisco, Rubisco activase, Rubisco activation, photosynthesis improvement, nitrogen, transgenic rice

## Abstract

It has been reported that overproduction of Rubisco activase (RCA) in rice (*Oryza sativa* L.) decreased Rubisco content, resulting in declining photosynthesis. We examined the effects of RCA levels on Rubisco content using transgenic rice with overexpressed or suppressed *RCA* under the control of different promoters of the *RCA* and Rubisco small subunit (*RBCS*) genes. All plants were grown hydroponically with different N concentrations (0.5, 2.0 and 8.0 mM-N). In RCA overproduced plants with > 2-fold RCA content (RCA-HI lines), a 10%–20% decrease in Rubisco content was observed at 0.5 and 2.0 mM-N. In contrast, at 8.0 mM-N, Rubisco content did not change in RCA-HI lines. Conversely, in plants with 50%–60% increased RCA content (RCA-MI lines), Rubisco levels remained unchanged, regardless of N concentration. Such effects on Rubisco content were independent of the promoter that was used. In plants with *RCA* suppression to < 10% of the wild-type RCA content, Rubisco levels were increased at 0.5 mM-N, but were unchanged at 2.0 and 8.0 mM-N. Thus, the effects of the changes in RCA levels on Rubisco content depended on N supply. Moreover, RCA overproduction was feasible without a decrease in Rubisco content, depending on the degree of RCA production.

## 1. Introduction

Ribulose-1,5-bisphosphate carboxylase/oxygenase (Rubisco), the enzyme that catalyzes the two competing reactions of CO_2_ fixation in photosynthesis and production of 2-phosphoglycolate in the photorespiratory pathway, is a rate-limiting factor for light-saturated photosynthesis at the present atmospheric air conditions [1,2]. Thus, Rubisco has been considered to be one of the most important targets for improving photosynthesis capacity (e.g., [3,4,5]). The activity of Rubisco is inhibited by the binding of sugar phosphates—ribulose-1,5-bisphosphate (RuBP, a substrate of Rubisco), carboxyarabinitol 1-phosphate (CA1P, a nocturnal metabolite), and xylulose-1,5-bisphosphate (XuBP) and 2,3-pentodiulose-1,5-bisphosphate (PDBP), which are “misfire” products of the multistep catalytic reactions—at the active site [6,7,8,9]. Rubisco activase (RCA) is a member of the AAA+ family of proteins [10] and mediates the activation of Rubisco by facilitating the removal of its inhibitors in an ATP-dependent manner [11,12,13,14]. Most plants (including rice) contain two isoforms of RCA generated by alternative splicing: a large isoform of 45–48 kDa and a small isoform of 41–43 kDa [11,15]. The large isoform is regulated by the ATP/ADP ratio and redox state in chloroplasts via two cysteine residues located in the C-terminal extension, whereas the small isoform is not regulated by redox [16,17]. In rice, the small isoform is more abundant than the large isoform [18,19].

To evaluate the effects of changes in RCA content on photosynthesis, transgenic plants with a reduced or increased RCA content were produced. As the activation state of Rubisco and the CO_2_ assimilation rate declined only when RCA content was reduced by > 60% (tobacco, [20,21,22]; Arabidopsis, [23]; rice, [24,25,26]), RCA content was considered to be in excess of steady-state photosynthesis. On the other hand, it has been suggested that the activation of Rubisco by RCA would be a limitation for photosynthesis under fluctuating light [23,26,27,28] and high temperature conditions [26,29,30]. Transgenic rice plants with overproduced RCA exhibited a higher activation state of Rubisco and faster photosynthesis induction when the plants were transferred from low to high light intensities [26,31]. However, in such RCA-overproduced rice, Rubisco content was decreased and, consequently, photosynthesis rates were also decreased [31]. Moreover, some studies have shown that Rubisco content was increased in plants with *RCA* suppression [20,24,32], while other studies reported that Rubisco content did not change in these plants [21,22,25,33]. Thus, the effects of RCA levels on Rubisco content in transgenic plants with RCA overproduction or suppression remain uncertain.

In the present study, the effects of RCA levels on Rubisco content were examined in transgenic rice with the overexpression or suppression of *RCA*. We reported previously a difference in the peak timing of gene expression between the Rubisco small subunit (*RBCS*) gene and *RCA* in rice plants: the mRNA levels of *RBCS* reached maxima during leaf expansion, while those of *RCA* reached maxima after the stage of full expansion [34]. In the RCA-overproduced rice plants generated by Fukayama et al. [31], *RCA* was overexpressed under the control of the promoter of the chlorophyll a/b-binding protein (*Cab*), which yields peak of gene expression during leaf expansion, similar to that observed for *RBCS* [35]. Considering these studies, it is possible that the overlap in the expression of the transgenic *RCA* gene with that of *RBCS* affects Rubisco biosynthesis. In addition, Fukayama et al. [32] investigated the manner in which the overexpression of *RCA* affects Rubisco content negatively and reported the possibility that the reduction in Rubisco content occurred at the Rubisco synthesis step. Therefore, in the present study, *RCA* was overexpressed under the control of the promoters of *RBCS* or *RCA* (Pro*_RBCS_* or Pro*_RCA_*). We generated several transgenic rice lines with different levels of RCA content: a > 2-fold, and 1.5-fold increase. In addition, we obtained transgenic plants with an RCA content that was < 10% of the wild-type counterpart. Nitrogen (N) is largely invested in chloroplasts as photosynthetic proteins [36]. Rubisco, the most abundant leaf protein, accounts for 10%–30% of total leaf N [37,38], and N allocation to Rubisco increases with increasing leaf N content in several species including rice [39,40]. Thus, we considered the possibility that the effects of RCA levels on Rubisco content were related to leaf N availability. Therefore, all transgenic lines were grown hydroponically with different N concentrations. The activation state of Rubisco and the rate of CO_2_ assimilation were also examined. Finally, we discussed whether it is possible to overproduce RCA without a decrease in Rubisco content and whether the overproduction of RCA leads to an improvement in photosynthesis.

## 2. Results

### 2.1. Transgenic Rice Plants with High Increase (HI) and Moderate Increase (MI) in RCA Content

**Figure 1:**
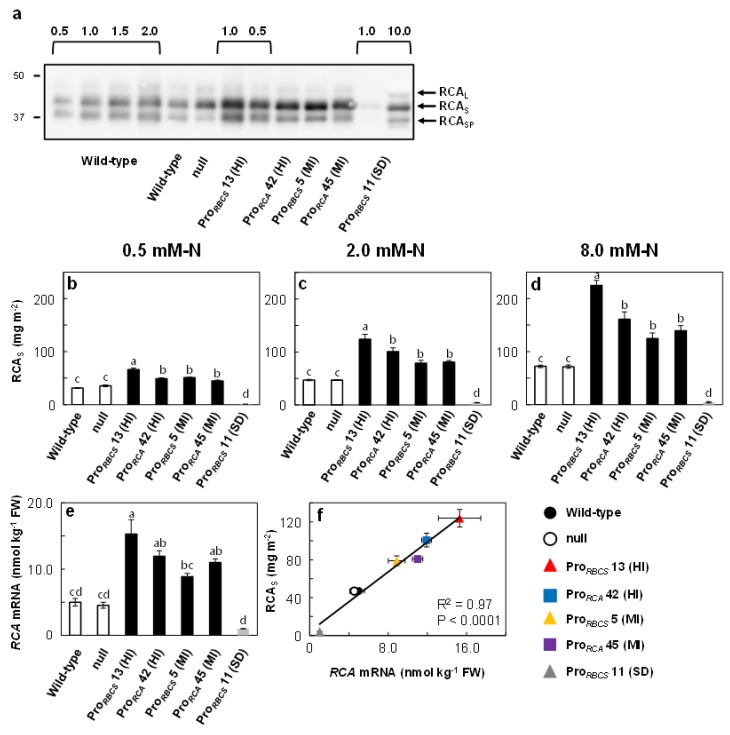
Detection of the Rubisco activase (RCA) protein and the *RCA* mRNA levels in RCA transgenic rice plants. (**a**) Detection of RCA in uppermost, fully expanded leaves at 2.0 mM-N concentration by Western blot analysis. Aliquots of SDS-treated samples at a volume corresponding to 0.1 μg of total leaf-N were subjected to SDS–PAGE. RCA was detected using specific antibodies after SDS–PAGE. The arrows indicate each isoform of RCA (large isoform of RCA (RCA_L_), small isoform of RCA (RCA_S_), and processed small isoform of RCA (RCA_SP_), described in [31]). (**b–d**) RCA_S_ content in a leaf area in uppermost, fully expanded leaves at 0.5, 2.0 and 8.0 mM-N concentrations. (**e**) *RCA* mRNA levels in expanding leaves of plants grown at 2.0 mM-N, on a tissue weight basis. The white, black, and grey bars indicate wild-type and null plants, RCA-overproduced plants, and plants with *RCA* suppression, respectively. (**f**) Relationship between RCA protein content and *RCA* mRNA level in plants grown at 2.0 mM-N. The linear regression line was calculated from datapoints using Pearson’s coefficient of correlation. The black circle, white circle, red triangle, blue square, yellow triangle, purple square, and grey triangle indicate wild-type, null, Pro*_RBCS_* 13 (HI), Pro*_RCA_* 42 (HI), Pro*_RBCS_* 5 (MI), Pro*_RCA_* 45 (MI), and Pro*_RBCS_* 11 (SD) plants, respectively. Data are means ± SE (*n* = 3–5). Statistical analysis was carried out by ANOVA with a post hoc Tukey–Kramer’s HSD test. Different letters indicate significant differences among genotypes (*p* < 0.05).

We transformed the cDNA of the small form of *RCA* (*Os11t0707000-2*) in the sense orientation under the control of the *RBCS* promoter (Pro*_RBCS_*) or of the *RCA* promoter (Pro*_RCA_*). We selected two lines with different levels of RCA accumulation for each type of transgenic rice plant: with a >2-fold increase in RCA content (high increase lines; HI) and with a 50% increase in RCA content (moderate increase lines; MI). These changes in RCA levels were independent of the promoter used. We also obtained another transgenic line with decreased RCA content, probably caused by a co-suppression effect (severe decrease line; SD). In Western blot analysis, three immunoreactive bands were detected. According to Fukayama et al. [31], these three bands correspond to different RCA isoforms: the large isoform (RCA_L_, top band), the small isoform (RCA_S_, middle band) and processed small isoform (RCA_SP_, bottom band) described in Vargas-Suárez et al. [41] (Figure 1a). The selected RCA increase or decrease lines, wild-type plants, and null segregants were grown hydroponically with three different N concentrations (0.5, 2.0, and 8.0 mM-N). The content of the small form of RCA (RCA_S_) was increased by 112%–212% and 57%–123% in Pro*_RBCS_* 13 (HI) and Pro*_RCA_* 42 (HI), and by 63%–73% and 43%–93% in Pro*_RBCS_* 5 (MI) and Pro*_RCA_* 45 (MI), respectively, relative to wild-type plants. In Pro*_RBCS_* 11 (SD), the RCA content was less than one-tenth of that detected in wild-type plants (Figure 1b-d). We also determined the mRNA levels of *RCA* by qRT-PCR in plants grown at 2.0 mM-N. The mRNA levels of *RCA* were increased by 205%, 138%, 77%, and 120% in Pro*_RBCS_* 13 (HI), Pro*_RCA_* 42 (HI), Pro*_RBCS_* 5 (MI), and Pro*_RCA_* 45 (MI), respectively, and decreased by 80% in Pro*_RBCS_* 11 (SD) (Figure 1e). *RCA* mRNA level was strongly correlated with RCA_S_ protein amount (Figure 1f).

### 2.2. Effects of Changes in RCA Content on Rubisco Levels in Plants Treated with Three Different N Concentrations

**Figure 2:**
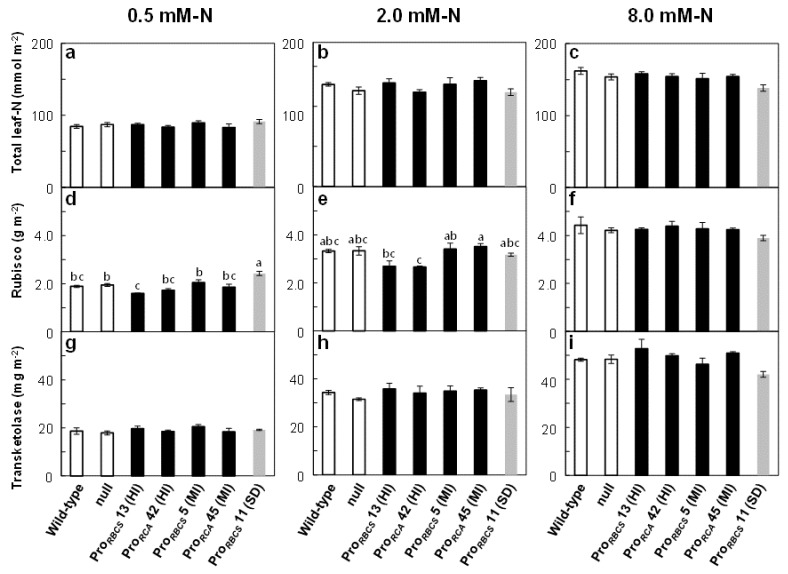
Total leaf-N, Rubisco, and transketolase contents in RCA transgenic plants. (**a–c**) Total leaf-N, (**d–f**) Rubisco, and (**e–i**) transketolase contents in a leaf area in uppermost, fully expanded leaves at 0.5, 2.0 and 8.0 mM-N concentrations. The white, black, and grey bars indicate wild-type and null plants, RCA-overproduced plants, and plants with *RCA* suppression, respectively. Data are means ± SE (*n* = 3–5). Statistical analysis was carried out by ANOVA with a post hoc Tukey–Kramer’s HSD test. Different letters indicate significant differences among genotypes (*p* < 0.05).

The total leaf N content did not differ among the genotypes for each N treatment (Figure 2a–c). In both RCA-HI lines, Rubisco content tended to decrease compared to the wild-type plants at 0.5 and 2.0 mM-N, but was not different at 8.0 mM-N. Rubisco contents of the two RCA-MI lines did not differ within the same N treatment. In Pro*_RBCS_* 11 (SD), Rubisco content was increased in the 0.5 mM-N treatment, but was not different at 2.0 and 8.0 mM-N (Figure 2d–f). These results suggest that, although the changes in RCA content tended to affect Rubisco levels negatively, these effects depended on the amount of N supply. Conversely, there were no differences in transketolase content among genotypes within the same N treatment (Figure 2g–i).

**Figure 3:**
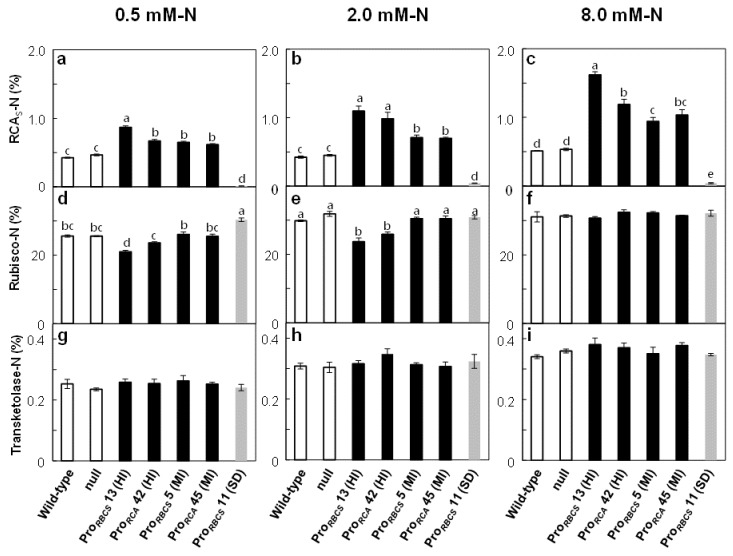
N allocation to RCA_S_, Rubisco, and transketolase in RCA transgenic plants. (**a–c**) Rubisco-N, (**d–f**) RCA_S_-N, and (**g–i**) transketolase-N in uppermost, fully expanded leaves at 0.5, 2.0 and 8.0 mM-N concentrations. The ratio of N contained in each protein to total leaf-N was estimated using a conversion factor of 0.16 for N to protein. The white, black, and grey bars indicate wild-type and null plants, RCA-overproduced plants, and plants with *RCA* suppression, respectively. Data are means ± SE (*n* = 3–5). Statistical analysis was carried out by ANOVA with a post hoc Tukey–Kramer’s HSD test. Different letters indicate significant differences among genotypes (*p* < 0.05).

Figure 3 shows the ratios of the N allocated to RCA, Rubisco, and transketolase to total leaf-N content in the three N concentrations. RCA_S_-N was increased by 105%–219%, 58%–133%, 53%–85%, and 45%–103% in Pro*_RBCS_* 13 (HI), Pro*_RCA_* 42 (HI), Pro*_RBCS_* 5 (MI), and Pro*_RCA_* 45 (MI) compared to the wild-type plants, and was greatly decreased in Pro*_RBCS_* 11 (SD) (to < 10%) (Figure 3a–c). In Pro*_RBCS_* 13 (HI) compared to wild-type plants, Rubisco-N was significantly decreased, by 18% in 0.5 mM-N and by 23% in 2.0 mM-N growth conditions. In Pro*_RCA_* 42 (HI) compared to wild-type, Rubisco-N was also decreased by 16% at 2.0 mM-N, and tended to decrease at 0.5 mM-N. On the other hand, Rubisco-N in Pro*_RBCS_* 13 (HI) and Pro*_RCA_* 42 (HI) was unchanged at 8.0 mM-N. Rubisco-N was increased by 19% in Pro*_RBCS_* 11 (SD) grown at 0.5 mM-N and was unchanged at 2.0 and 8.0 mM-N. In contrast, Rubisco-N was unchanged in the two RCA-MI lines at any N concentration (Figure 3d–f). On the other hand, there was no difference in transketolase-N, regardless of genotype (Figure 3g–i). As the increase or decrease in N allocation to RCA by transgenesis corresponded to only < 1% of the total leaf-N content, the changes in Rubisco content observed in transgenic lines were not explained by the changes in the amount of RCA.

**Figure 4:**
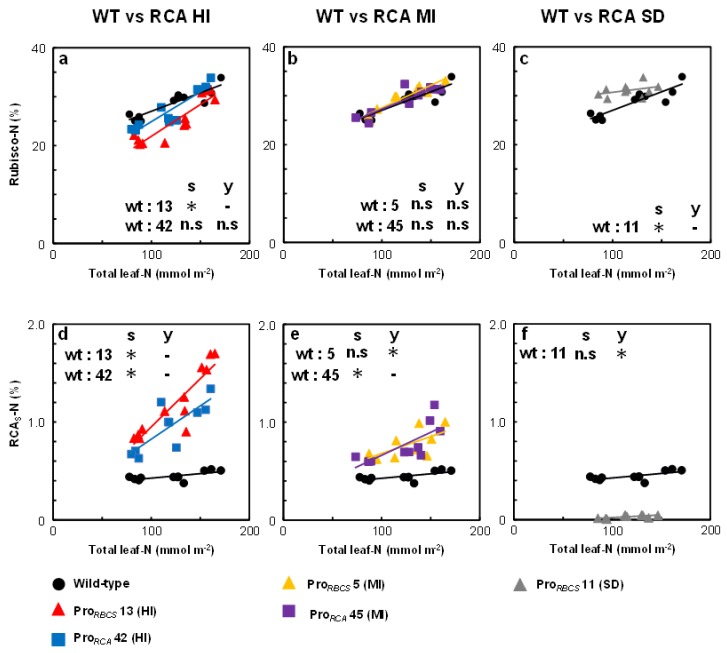
Relationships between Rubisco-N and RCA_S_-N and total leaf-N contents in RCA transgenic plants. The data were taken from Figure 2 and Figure 3. (**a–c**) Relationships between Rubisco-N and total leaf-N and (**d–f**) RCA_S_-N and total leaf-N in uppermost, fully expanded leaves. The black circle, red triangle, blue square, yellow triangle, purple square, and grey triangle indicate wild-type, Pro*_RBCS_* 13 (HI), Pro*_RCA_* 42 (HI), Pro*_RBCS_* 5 (MI), Pro*_RCA_* 45 (MI), and Pro*_RBCS_* 11 (SD) plants, respectively. The asterisks denote a statistically significant difference, as assessed by analysis of covariance (ANCOVA) (*p* < 0.05) performed on the slope (s) and y-intercept (y) of the linear regressions between wild-type and RCA transgenic plants, respectively. The details of the statistical treatments are presented in Tables S1 and S2.

The relationships between Rubisco-N, RCA_S_-N, and total leaf-N contents were examined (Figure 4). The regression plots between Rubisco-N and total leaf-N content showed that the slope in Pro*_RBCS_* 13 (HI) was significantly steeper, and the slope in Pro*_RCA_* 42 (HI) was slightly steeper, than that observed in wild-type plants. In the two RCA-MI lines, the regression slopes were similar to those detected in wild-type plants. Conversely, the slope in Pro*_RBCS_* 11 (SD) was significantly slower vs. the wild-type one. The differences in Rubisco-N between RCA transgenic and wild-type plants decreased with increasing total leaf-N content: at the maximum total leaf-N content, Rubisco-N converged to 30% of total leaf-N, regardless of genotype (Figure 4a–c). It was reported that a value of 30% in Rubisco-N is close to maximum levels in rice plants [4,38,42,43,44]. These results clearly showed that the effects of the changes in RCA content on Rubisco levels depended on total leaf-N content. The regression plots between RCA_S_-N and total leaf-N content showed that the slopes in the RCA-HI and Pro*_RCA_* 45 (MI) lines were steeper, and the slope in Pro*_RBCS_* 5 (MI) was slightly steeper, than that observed for the wild-type plants (Figure 4d–f). Thus, the increase in N allocation to RCA by transgenesis was augmented by increasing the total leaf-N content.

### 2.3. Activation State of Rubisco and the Rate of CO_2_ Assimilation in Transgenic Rice Plants 

**Figure 5:**
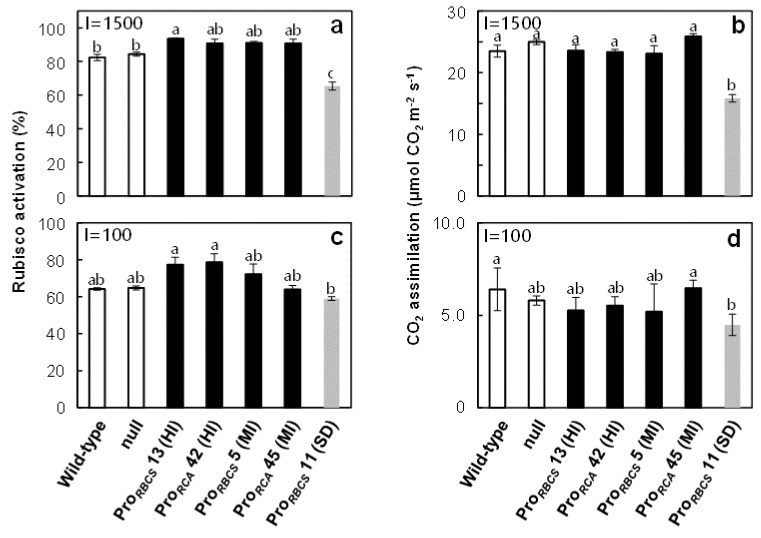
(**a,c**) Rubisco activation and (**b,d**) the rates of CO_2_ assimilation at an ambient CO_2_ partial pressure (Ca = 40 Pa) and high and low irradiances (PPFD of 1500 and 100 μmol quanta m^–2^ s^–1^). Measurement were done on uppermost, fully expanded leaves in plants grown at 2.0 mM-N. The white, black, and grey bars indicate wild-type and null plants, RCA-overproduced plants, and plants with *RCA* suppression, respectively. Data are means ± SE (*n* = 3–5). Statistical analysis was carried out by ANOVA with a post hoc Tukey–Kramer’s HSD test. Different letters indicate significant differences among genotypes (*p* < 0.05).

To examine the effects of overproduction of RCA on photosynthesis, the activation state of Rubisco and the rate of CO_2_ assimilation under conditions of ambient [CO_2_] partial pressures (Ca = 40 Pa) and high and low irradiances (PPFD of 1500 and 100 μmol quanta m^–2^ s^–1^) were measured in plants grown at 2.0 mM-N (Figure 5). Under high irradiance conditions, the activation state of Rubisco in Pro*_RBCS_* 13 (HI) was significantly higher, and the activation state of Rubisco in other RCA overproduced plants tended to be higher than that in wild-type plants (Figure 5a). However, the rate of CO_2_ assimilation did not increase in RCA-overproduced plants compared to wild-type plants (Figure 5b). Conversely, the activation state of Rubisco and the rate of CO_2_ assimilation in Pro*_RBCS_* 11 (SD) were significantly lower than in wild-type plants. Similar results were reported in previous studies with *RCA*-suppressed rice plants [24,25]. Under low irradiance conditions, although the activation state of Rubisco in RCA-overproduced plants also tended to be higher than in wild-type plants, there was no increase in the rate of CO_2_ assimilation (Figure 5c,d).

## 3. Discussion

Many studies have shown that RCA and Rubisco are attractive targets for improving photosynthesis (for a review, see [4]). However, Fukayama et al. [31,32] reported that the overproduction of RCA negatively affected Rubisco content. To assess whether it is possible to overproduce RCA without affecting Rubisco content, we generated and selected several transgenic rice plants exhibiting varying RCA levels under the control of two different promoters (Figure 1) and examined the effects of changes in RCA levels on Rubisco content in plants grown with different N concentrations. Our results showed that although changes in RCA levels tended to affect Rubisco content negatively, these effects depended on N supply and were diminished with increasing total leaf-N content (Figure 2, Figure 3 and Figure 4). In addition, Rubisco levels were unchanged in transgenic rice plants, with a 50% increase in RCA content regardless of N concentration (Figure 2, Figure 3 and Figure 4). These results showed that overproduction of RCA without a decrease in Rubisco content was feasible via a moderate increase in RCA levels. The effects of changes in RCA content on Rubisco levels are discordant between previous studies of rice plants with *RCA* suppression: Jin et al. [24] showed that Rubisco content was increased, whereas Masumoto et al. [25] did not find such an increase in Rubisco content in those plants. Our results (Figure 2, Figure 3 and Figure 4) indicate the possibility that the differences observed between the studies of Jin et al. [24] and Masumoto et al. [25] were caused by differences in N availability (summarized in Table S3). We observed a significant increase in Rubisco content exclusively in the low-N-grown *RCA*-suppressed plants (Figure 2). 

Previously, we found a negative correlation between the amounts of Rubisco and RCA in transgenic rice plants with increased or decreased Rubisco content. However, such a negative correlation was also observed for enzymes of the Calvin–Benson cycle. Therefore, these phenomena were non-specific effects that were accounted for by changes in N allocation caused by changes in Rubisco content [45]. In contrast, in the present study of RCA-transgenic plants, the amounts of transketolase and transketolase-N were not affected (Figure 2 and Figure 3). Similarly, Fukayama et al. [32] reported no changes in several Calvin–Benson cycle enzymes in RCA-transgenic rice plants. These results suggest that the changes in RCA content affected Rubisco levels selectively. In addition, as changes in N allocation to RCA_S_ in RCA-transgenic plants corresponded to less than 1% of the total leaf-N content (Figure 3), the changes in Rubisco content observed in transgenic plants were not explained by the changes in the amount of RCA. Therefore, although we observed a negative correlation between Rubisco and RCA content when the amounts of RCA or Rubisco were genetically manipulated, the effects of changes in RCA content on Rubisco levels in RCA-transgenic plants were different from those detected in Rubisco-transgenic plants.

Our results showed that although the levels of the Rubisco protein were decreased in RCA-HI plants, the mRNA levels of *RBCS* and of the Rubisco large subunit (*RBCL*) gene remained unchanged (Figure S1). Similar results were obtained for the *RCA*-overexpressing rice plants reported by Fukayama et al. [31,32]. These results suggest that Rubisco content is regulated post-transcriptionally. It has been suggested that the unassembled RBCL protein interacts with the *RBCL* mRNA, leading to a decline in its polysome loading and the suppression of its translation in tobacco plants [46,47]. This assembly-dependent translational regulation mechanism is termed control by epistasy of synthesis (CES) [48]. In our previous studies of rice plants [49,50], the amount of RBCL loading on polysomes was decreased in senescent leaves, although regulation at the transcription level principally controls RBCL protein synthesis. This suggests that CES regulation is also effective in controlling RBCL synthesis in a senescent rice leaf. Although Fukayama et al. [32] reported the possibility that the translation activities of *RBCS* and *RBCL* did not greatly change in *RCA*-overexpressing rice plants, it is unclear whether the small decline in Rubisco content observed in RCA-overproduced rice plants was caused by CES regulation of *RBCL*.

We reported previously that the mRNA levels of *RBCS* reached maxima during leaf expansion, followed by a rapid decrease, while those of *RCA* reached maxima after the stage of full expansion to senescence [34]. In addition, based on the argument that RCA affects Rubisco synthesis negatively, described in Fukayama et al. [32], we considered the possibility that the overlap in the expression of the *RCA* transgene with that of the *RBCS* gene affects Rubisco biosynthesis. Therefore, we used Pro*_RBCS_* and Pro*_RCA_* for the overproduction of RCA. However, Rubisco content decreased in RCA-HI plants and did not decrease in RCA-MI plants, regardless of the promoter that was used (Figure 2 and Figure 3). Thus, Rubisco content was not affected by the difference in two promoters used for RCA overproduction, but by the magnitude of overproduction of RCA.

The activation state of Rubisco tended to increase in RCA-overproduced plants (Figure 5), which is consistent with the previous study [31]. It was considered that the enhancement of Rubisco activation compensated for the decrease in Rubisco content in RCA-HI plants. On the other hand, in RCA-MI plants, the effects of enhancement of Rubisco activation on the rate of CO_2_ assimilation were not observed (Figure 5). This is probably because under ambient [CO_2_] conditions, the rate of CO_2_ assimilation was mainly limited by Rubisco capacity, but also partly limited by RuBP regeneration capacity. Our previous studies showed that the overproduction of Rubisco did not necessarily enhance photosynthesis because of decreased Rubisco activation, probably due to a decrease in RCA content [45,51,52,53]. Thus, we considered that overproduction of both Rubisco and RCA is essential to improve photosynthesis, especially under low [CO_2_] conditions. On calculation, an increase in RCA content of 50%–60% in RCA-MI plants is considered to be sufficient to compensate for the decrease in RCA levels caused by Rubisco overproduction. In a future study, we will attempt to generate transgenic rice plants with co-overproduction of Rubisco and RCA, to enhance the photosynthetic capacity. 

## 4. Materials and Methods 

### 4.1. Generation of Transgenic Plants

The binary vector pBIRS [51] was digested with *Hind*III and *Sac*I. A DNA fragment of the region corresponding to –3000 to +36 from the start codon of the small form of *RCA* [*Os11t0707000-2*; RAP-DB [54]] was amplified as its promoter region (Pro*_RCA_*) by genomic PCR using PrimeSTAR HS (TAKARA, Shiga, Japan) and the following primer pair: 5’–TGATTACGCCAAGCTTAATGCTTGAAATATAATGCTGCG–3’ and 5’–CGGAGCTCCAACGGTGGAG–3’. A DNA fragment from position +22 from the *RCA* start codon to its end was amplified from the cDNA template of rice (*Oryza sativa* L. cv Notohikari) by PrimeSTAR HS with the following primer pair: 5’–ACCGTTGGAGCTCCGGCGT–3’ and 5’–GATCGGGGAAATTCGAGCTCAATGAAATATACTCATGTATAGTAT–3’. A DNA fragment containing the open reading frame (ORF) and the 5’ untranslated region (5’-UTR) of *RCA* was amplified. A cDNA template was amplified by reverse-transcription PCR (RT–PCR) using SuperScript® III Reverse Transcriptase (Thermo Fisher Scientific, Yokohama, Japan). The digested pBIRS and the amplified DNA fragment were fused using an In-Fusion HD Cloning Kit (Mountain View, Clontech, CA, USA) according to the manufacturer’s instructions, to generate a vector for the overexpression of *RCA* under the control of its own promoter (Pro*_RCA_*). The *RBCS* promoter (Pro*_RBCS_*) (2.8 kb fragment of the rice *RBCS* promoter, as described by Kyozuka et al. [55]) was amplified using PrimeSTAR HS and the following primer pair: 5’–TGATTACGCCAAGCTTGCATGCCT–3’ and 5’–CGGCCGCTGCTGCTCAAGCTTATCGATACCGTCGAC–3’. A DNA fragment corresponding to positions –62 from the *RCA* start codon to its end was amplified from the cDNA template by primeSTAR HS using the following primer pair: 5’–GAGCAGCAGCGGCCGGC–3’ and 5’–GATCGGGGAAATTCGAGCTCAATGAAATATACTCATGTATAGTAT–3’. The digested pBIRS and the amplified DNA fragment were fused using an In-Fusion HD Cloning Kit to generate a vector for the overexpression of *RCA* under the control of the *RBCS* promoter (Pro*_RBCS_*).

Rice (*Oryza sativa* L. cv Notohikari) was transformed using the *agrobacterium* method [56]. The T_0_ progenies of transgenic plants were grown hydroponically in an isolated greenhouse [2]. Transgenic plants with an increase in RCA content were screened and self-fertilized to collect T_1_ seeds. Selections of homozygotes were performed using the comparative cycle threshold method, as described by Suzuki et al. [57]. The primer pairs used in this experiment were as follows: 5’–CAATTTCACACAGGAAACAGCTATG–3’ and 5’–TTTGCTGCAGCATGCA–3’ for Pro*_RBCS_ RCA*-overexpressing (ox) plants and 5’–GGATAACAATTTCACACAGGAAACA–3’ and 5’–CGCAGCATTATATTTCAAGCATTAA–3’ for Pro*_RCA_ RCA* ox plants. The probes used were as follows: reporter, FAM; quencher, NFQ-MGB; 5’–CCATGATTACGCCAAGC–3’ for Pro*_RBCS_ RCA* ox plants and 5’–CTATGACCATGATTACGC–3’ for Pro*_RCA_ RCA* ox plants. The selected T_1_ progenies were allowed to self-fertilize, to collect T_2_ seeds. We also screened transgenic plants with a decrease in RCA content caused by co-suppression and collected T_2_ seeds, as described above. Null segregants were also selected and self-fertilized to collect T_2_ seeds. Two lines each of the T_2_ progenies of Pro*_RBCS_ RCA* ox plants and Pro*_RCA_ RCA* ox plants and one line with *RCA* suppression were used in this study. Both null segregant lines derived from Pro*_RBCS_* transgenic plants and from Pro*_RCA_* transgenic plants were used. The data from all null segregant lines were aggregated into a single “null”, used for analysis. To confirm that both null segregant lines showed trends similar to wild-type plants, segregants lines derived from Pro*_RBCS_* plants or Pro*_RCA_* plants were grouped and analyzed separately in Figure S2. Relationships of total leaf-N with RCA content and total leaf-N with Rubisco content did not differ between wild-type and the two null segregant lines.

### 4.2. Plant Culture and Sampling

Plants were grown hydroponically in an environmentally controlled growth chamber, as described by Suganami et al. [45], with slight modifications. The growth chamber was operated with a PPFD of 800 μmol quanta m^−2^ s^−1^, a 15 h photoperiod and a day/night temperature of 26/20 °C. From the 63rd to 77th day after sowing, the uppermost, fully expanded leaves were collected after measurement of the rate of CO_2_ assimilation and stored at –80 °C until biochemical assays. For the determination of mRNA levels, leaves that had emerged from their sheaths by 60% were collected. Samplings were done between 11:00 and 13:00, from the 4th to 7th day after renewal of the nutrient solution. 

### 4.3. Measurement of Gas Exchange

Measurements of the CO_2_ assimilation rate were performed using a portable gas exchange system (LI-6400XT, Li-Cor, Lincoln, NE, USA). Conditions in the chamber were as follows: leaf temperature of 25 °C, Ca of 40 Pa, leaf-to-air vapor pressure difference of 1.0–1.2 kPa, relative humidity of 60%–70%, PPFD of 1500 or 100 μmol quanta m^−2^ s^−1^. The steady state of gas exchange rate was obtained. To compute CO_2_ assimilation rate, gas exchange parameters were calculated according to equations of von Caemmerer and Farquhar [58].

### 4.4. Biochemical Assay

The amounts of total leaf-N, Rubisco, small form of RCA (RCA_S_) and transketolase were determined on the same leaf. The frozen leaves were homogenized in Na-phosphate buffer (pH 7.0) containing 2 mM iodoacetic acid, 120 mM 2-mercaptoethanol, and 5% (v/v) glycerol. The total N content in leaves was determined using Nessler’s reagent after Kjeldahl digestion [42]. Rubisco content was determined spectrophotometrically after formamide extraction of Coomassie Brilliant Blue (CBB) R-250-stained bands corresponding to the large and small subunits of Rubisco separated by SDS–PAGE. A calibration curve was prepared with Rubisco purified from rice [59]. RCA_S_ and transketolase contents were determined by image analysis after SDS–PAGE followed by CBB G-250 staining, as described by Suganami et al. [45], with slight modification. Exceptionally, because RCA content in Pro*_RBCS_* SD plants could not be quantified via CBB staining, Western blot analyses were used to quantify the protein in these plants, as described by Suzuki et al. [57]. Antibodies against RCA were purchased from Agrisera (Vännäs, Sweden). For the calculation of N allocation to Rubisco, RCA_S_ and transketolase, a nitrogen to protein conversion factor of 0.16 was used.

Rubisco activity was measured spectrophotometrically by coupling 3-phosphoglyceric acid formation with NADH oxidation at 25 °C, according to [60], with slight modification. The samples used for the Rubisco activation assay were collected from a leaf that was equilibrated at steady-state conditions. After exposure to at least 30 min of illumination (PPFD of 1500 or 100 μmol quanta m^–2^ s^–1^) in the chamber of the portable gas exchange system (LI-6400XT, Li-Cor) and after gas exchange had reached the steady-state rate (leaf temperature of 25 °C, Ca of 40 Pa), the leaf was quickly frozen in liquid N_2_. The frozen leaf was quickly (within 30 s) homogenized in 50 mM HEPES/NaOH (pH 8.0) containing 20 mM MgCl_2_, 10 mM dithiothreitol, and the protease inhibitor cocktail Complete Mini (Roche, Manheim, Germany) using an ice-chilled mortar and pestle. After centrifugation at 4 °C for 10 s, a portion of the supernatant was injected into a reaction mixture of 100 mM HEPES/NaOH (pH 8.0) containing 20 mM MgCl_2_, 5 mM dithiothreitol, 5 mM ATP, 5 mM phosphocreatine, 0.2 mM NADH, 100 mM NaHCO_3_, 0.5 mM ribulose 1,5-bisphosphate (RuBP), 25 U mL^–1^ of glyceraldehyde-3-phosphate dehydrogenase, 25 U mL^–1^ of 3-phosphoglycerate kinase, and 25 U mL^–1^ of creatine phosphokinase. Total activity was measured in the supernatant after incubation with 100 mM NaHCO_3_ and 20 mM MgCl_2_. The activation state was taken as the ratio of the initial activity to the total activity.

### 4.5. RNA Analysis

Analyses of mRNA levels were done on expanding leaves of plants grown at 2.0 mM-N. Total RNA was extracted according to the method of Suzuki et al. [61], with slight modification [53]. The mRNA levels were determined by quantitative reverse transcription PCR (qRT–PCR) according to Ogawa et al. [62]. The used primer pairs for *RCA* can be found in Yamaoka et al. [34] and those for *RBCS* and *RBCL* can be found in Suzuki et al. [53].

### 4.6. Statistical Treatments

Data are means ± SE. Data shown in Figure 1, Figure 2 and Figure 3, Figure 5, and Figure S1 were statistically analyzed with ANOVA followed by the post hoc Tukey–Kramer’s HSD test (*p* < 0.05). First, one-way ANOVA was used to test for statistically significant differences between means of each trait among the seven genotypes. If a significant difference was found, post hoc Tukey–Kramer’s HSD test was carried out for multiple pairwise comparisons. The relationships between datasets in Figure 1f, Figure 4, and Figure S2 were evaluated using Pearson’s correlation coefficient. Data shown in Figure 4 were statistically analyzed by analysis of covariance (ANCOVA). First, the slopes of regression line were tested; if no significant difference was found, intercepts were then tested. All analyses were carried out using JMP11 (SAS Institute Japan, Tokyo, Japan).

## Supplementary Materials

Supplementary Materials can be found at https://www.mdpi.com/1422-0067/21/5/1626/s1.

## Abbreviations

CES	control by epistasy of synthesis
HI	high increase
MI	moderate increase
Pro*_RBCS_*	the promoter of *RBCS*
Pro*_RCA_*	the promoter of *RCA*
RBCL	large subunit of Rubisco
RBCS	small subunit of Rubisco
RCA	Rubisco activase
RuBP	Ribulose 1,5-bisphosphate
SD	severe decrease
SE	standard error
