# Supplementary material for: Effects of Overproduction of Rubisco Activase on Rubisco Content in Transgenic Rice Grown at Different N Levels
Source: Int J Mol Sci. 2020 Feb 27;21(5):1626. doi: 10.3390/ijms21051626 (PMC7084177; doi:10.3390/ijms21051626)
Supplement: Supplementary file 1 [file ijms-21-01626-s001.pdf]

Supplemental data

Effects of overproduction of Rubisco activase on Rubisco content in transgenic rice grown at different N levels

List of materials:

Figure S1 Total RCA content and the mRNA levels of *RBCS* and *RBCL* in RCA-transgenic plants.

Figure S2 Relationships between total leaf-N and Rubisco and RCA content in wild-type and null segregants, derived from Pro$_{RBCS}$ plants and Pro$_{RCA}$ plants.

Table S1 Statistical analysis of the regression lines between Rubisco-N and total leaf-N content.

Table S2 Statistical analysis of the regression lines between RCA$_S$-N and total leaf-N content.

Table S3 Comparison of previous studies and our present study.

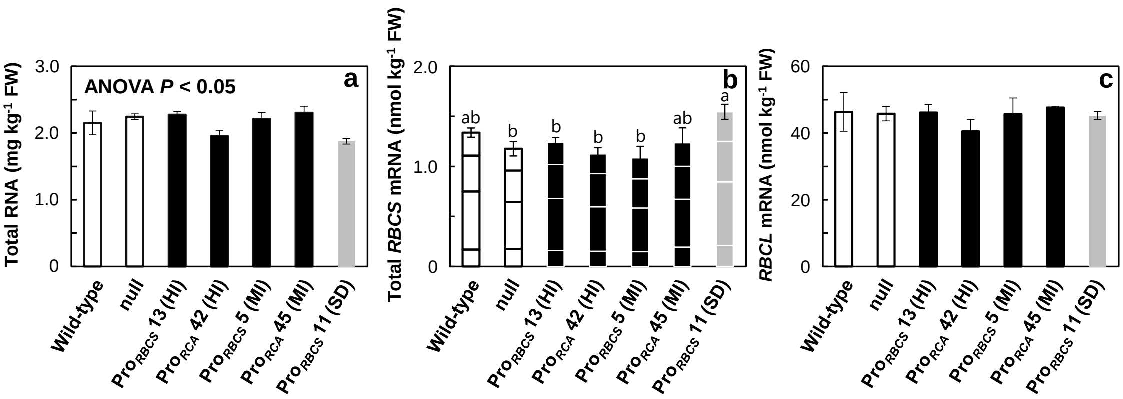

Figure S1 mRNA levels of total *RBCS*, *RBCL* and *RCA* and total RNA content in RCA transgenic plants. (a) total RNA content and mRNA levels of (b) total *RBCS* and (c) *RBCL* in expanding leaves on a tissue weight basis. In total *RBCS*, each bar was divided into four parts, which indicate the mRNA levels of four genes of *RBCS* multigene family, *RBCS2*, *3*, *4* and *5*, respectively, from the bottom to top. The white, black and grey bars indicate wild-type and null plants, RCA-overproduced plants and plants with *RCA* suppression, respectively. Data are means ± SE (n = 3–4). Statistical analysis was carried out by ANOVA with a post hoc Tukey-Kramer's HSD test. Different letters indicate significant differences among the genotypes ($P < 0.05$).

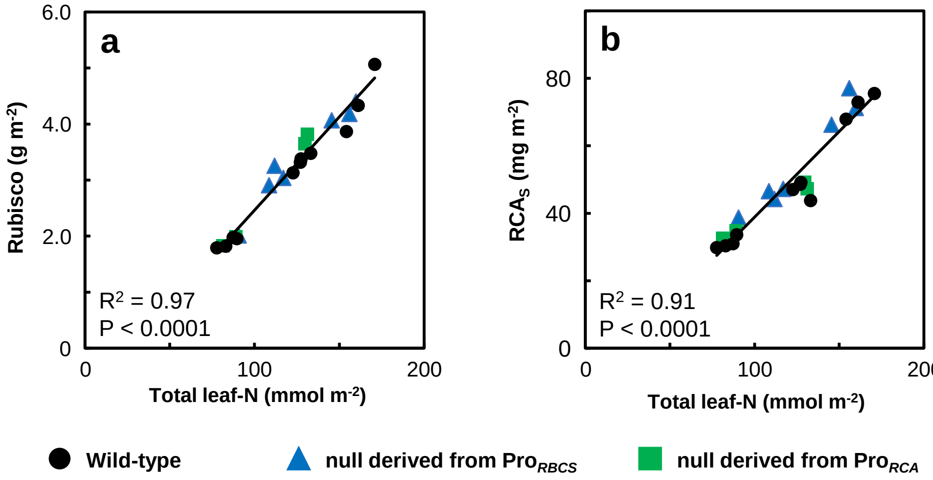

Figure S2 Relationships between total leaf-N and Rubisco and RCA content in wild-type and null segregants, derived from Pro*RBCS* plants and Pro*RCA* plants. (a) Relationships between Rubisco and total leaf-N and (b) RCA and total leaf-N content in uppermost, fully expanded leaves. The linear regression line was calculated from data points using Pearson's coefficient of correlation. The black circle, blue triangle and green square indicate wild-type, null segregant line derived from Pro*RBCS* plants and null segregant line derived from Pro*RCA* transgenic plants.

Table S1 Statistical analysis of the regression lines between Rubisco-N and total leaf-N content. Data are presented in Figure 4. The linear regression line was calculated from the data points using Pearson's coefficient of correlation. Analysis of covariance (ANCOVA) was performed on the slope and y-intercept of the linear regressions between wild-type and RCA transgenic plants, respectively. Valuation were based on the results of test performed at $P < 0.05$.

**Line**	**Regression Formulae**	**P value (slope)**		**P value (Y-intercept)**		**Valuation**
**wild-type**	y = 0.0758 x + 19.5, (r^2 = 0.81, P = 0.0002)	-	-	-	-	-
**Pro$_{RBCS}$ 13 (HI)**	y = 0.130 x + 8.9, (r^2 = 0.82, P < 0.0001)	0.028	*	<0.0001	-,**	Intersect
**Pro$_{RCA}$ 42 (HI)**	y = 0.122 x + 12.7, (r^2 = 0.83, P = 0.0002)	0.053	n.s	0.103	n.s	Same line
**Pro$_{RBCS}$ 5 (MI)**	y = 0.093 x + 18.3, (r^2 = 0.90, P < 0.0001)	0.346	n.s	0.090	n.s	Same line
**Pro$_{RCA}$ 45 (MI)**	y = 0.082 x + 19.1, (r^2 = 0.78, P = 0.0007)	0.759	n.s	0.499	n.s	Same line
**Pro$_{RBCS}$ 11 (SD)**	y = 0.025 x + 28.2, (r^2 = 0.16, P = 0.2559)	0.049	*	0.0002	-,**	Intersect

* means $P < 0.05$
** means P corrected after Bonferroni correction for multiple test, $P < 0.01$

Table S2 Statistical analysis of the regression lines between RCA$_S$-N and total leaf-N content. Data are presented in Figure 4. The linear regression line was calculated from the data points using Pearson's coefficient of correlation. Analysis of covariance (ANCOVA) was performed on the slope and y-intercept of the linear regressions between wild-type and RCA transgenic plants, respectively. Valuation were based on the results of test performed at $P < 0.05$.

**Line**	**Regression Formulae**	**P value (slope)**		**P value (Y-intercept)**		**Valuation**
**wild-type**	y = 0.000918 x + 0.335 (r^2 = 0.47, P = 0.0208)	-	-	-	-	-
**Pro$_{RBCS}$ 13 (HI)**	y = 0.00985 x – 0.034 (r^2 = 0.81, P < 0.0001)	<0.0001	**	-	-	Intersect
**Pro$_{RCA}$ 42 (HI)**	y = 0.006805 x + 0.145 (r^2 = 0.64, P = 0.0053)	0.0024	**	-	-	Intersect
**Pro$_{RBCS}$ 5 (MI)**	y = 0.003468 x + 0.333 (r^2 = 0.46, P = 0.0317)	0.0546	n.s	<0.0001	**	Parallel line
**Pro$_{RCA}$ 45 (MI)**	y = 0.004696 x + 0.192 (r^2 = 0.54, P = 0.0151)	0.0177	*	<0.0001	-,**	Intersect
**Pro$_{RBCS}$ 11 (SD)**	y = 0.000534 x – 0.031 (r^2 = 0.44, P = 0.0357)	0.4543	n.s	<0.0001	**	Parallel line

* means $P < 0.05$
** means *P* corrected after Bonferroni correction for multiple test, $P < 0.01$

Table S3 Comparison of previous studies and our present study. Data were cited from Table 1 in Jin et al. [24], Table 1 in Masumoto et al. [25] and Figures 2 and 3 in the present study.

Jin et al. (2006)	Total leaf-N content (mmol m^{-2})	Rubisco content (g m^{-2})	Rubisco-N (%)
**wild-type**	n.d	1.68 ± 0.20 [b]	n.d
***RCA*-sup (30% of WT level)**	n.d	2.97 ± 0.57 [a]	n.d

Data are presented as means ± SE (n=6). Different letters indicate statistical difference at $p < 0.05$ (Student's *t*-test). Total leaf-N content and Rubisco-N were not determined.

Present study (0.5 mM-N)	Total leaf-N content (mmol m^{-2})	Rubisco content (g m^{-2})	Rubisco-N (%)
**Wild-type**	84.3 ± 2.6 [a]	1.89 ± 0.05 [b]	25.6 ± 0.3 [b]
***RCA*-sup (less than 10%)**	91.3 ± 2.9 [a]	2.42 ± 0.09 [a]	30.3 ± 0.6 [a]

Data are presented as means ± SE (n=3-4). Different letters indicate statistical difference at $p < 0.05$ (Student's *t*-test).

Masumoto et al. (2012)	Total leaf-N content (mmol m^{-2})	Rubisco content (g m^{-2})	Rubisco-N (%)
**Wild-type**	116.4 ± 15.7 [a]	2.96 ± 0.31 [a]	29.1 (calculated)
***RCA*-sup (AM) (20-25%)**	115.0 ± 24.3 [a]	3.43 ± 0.37 [a]	34.1 (calculated)
***RCA*-sup (AS) (5-15%)**	110.7 ± 7.1 [a]	3.09 ± 0.34 [a]	31.9 (calculated)

Data are presented as means ± SD (n=5-8). Different letters indicate statistical difference at $p < 0.05$ (Tukey-Kramer multiple comparison test). Since Rubisco-N was not present in original paper, we calculated.

Present study (2.0 mM-N)	Total leaf-N content (mmol m^{-2})	Rubisco content (g m^{-2})	Rubisco-N (%)
**Wild-type**	127.5 ± 2.2 [a]	3.33 ± 0.07 [a]	29.8 ± 0.2 [a]
***RCA*-sup (less than 10%)**	117.7 ± 4.1 [a]	3.17 ± 0.06 [a]	30.9 ± 0.5 [a]

Data are presented as means ± SE (n=4). Different letters indicate statistical difference at $p < 0.05$ (Student's *t*-test).
